# Mucosal-Associated Invariant T Cells Expressing the TRAV1-TRAJ33 Chain Are Present in Pigs

**DOI:** 10.3389/fimmu.2019.02070

**Published:** 2019-09-03

**Authors:** Xingxing Xiao, Kun Li, Xueting Ma, Baohong Liu, Xueyang He, Shunli Yang, Wenqing Wang, Baoyu Jiang, Jianping Cai

**Affiliations:** ^1^State Key Laboratory of Veterinary Etiological Biology, Key Laboratory of Veterinary Parasitology of Gansu Province, Lanzhou Veterinary Research Institute, Chinese Academy of Agricultural Sciences, Lanzhou, China; ^2^Jiangsu Co-innovation Center for Prevention and Control of Animal Infectious Diseases and Zoonoses, Yangzhou, China; ^3^State Key Laboratory of Veterinary Etiological Biology, Lanzhou Veterinary Research Institute, Chinese Academy of Agricultural Sciences, Lanzhou, China

**Keywords:** pigs, immunity, T cell receptors, mucosal-associated invariant T cells, phenotype

## Abstract

Mucosal-associated invariant T (MAIT) cells are a subpopulation of evolutionarily conserved innate-like T lymphocytes bearing invariant or semi-invariant TCRα chains paired with a biased usage of TCRβ chains and restricted by highly conserved monomorphic MHC class I-like molecule, MR1. Consistent with their phylogenetically conserved characteristics, MAIT cells have been implicated in host immune responses to microbial infections and non-infectious diseases, such as tuberculosis, typhoid fever, and multiple sclerosis. To date, MAIT cells have been identified in humans, mice, cows, sheep, and several non-human primates, but not in pigs. Here, we cloned porcine MAIT (pMAIT) TCRα sequences from PBMC cDNA, and then analyzed the TCRβ usage of pMAIT cells expressing the TRAV1-TRAJ33 chain, finding that pMAIT cells use a limited array of TCRβ chains (predominantly TRBV20S and TRBV29S). We estimated the frequency of TRAV1-TRAJ33 transcripts in peripheral blood and tissues, demonstrating that TRAV1-TRAJ33 transcripts are expressed in all tested tissues. Analysis of the expression of TRAV1-TRAJ33 transcripts in three T-cell subpopulations from peripheral blood and tissues showed that TRAV1-TRAJ33 transcripts can be expressed by CD4^+^CD8^−^, CD8^+^CD4^−^, and CD4^−^CD8^−^ T cells. Using a single-cell PCR assay, we demonstrated that pMAIT cells with the TRAV1-TRAJ33 chain express cell surface markers IL-18Rα, IL-7Rα, CCR9, CCR5, and/or CXCR6, and transcription factors PLZF, and T-bet and/or RORγt. In conclusion, pMAIT cells expressing the TRAV1-TRAJ33 chain have characteristics similar to human and mouse MAIT cells, further supporting the idea that the pig is an animal model for investigating MAIT cell functions in human disease.

## Introduction

T lymphocytes, consisting of conventional and unconventional T cells, play vital roles in immune responses. The two arms of the immune response, the innate and adaptive immune systems, which are distinct but interacting, respond to invading pathogens through innate immune cells or conventional B and T cells, respectively (1–3). Apart from these effector cells, there is an additional group of T cells that have both innate and adaptive properties, known as unconventional or innate-like T cells (2–4). These cells recognize non-peptide antigens presented by non-polymorphic major histocompatibility complex (MHC) molecules, and have larger clonal sizes than conventional T cells (2, 3, 5). There are two distinct subsets of innate-like T cells with a semi-invariant αβ TCR that have potential roles in combating microbial infections and chronic diseases. Invariant natural killer T (iNKT) cells constitute one subset, and mucosal-associated invariant T (MAIT) cells are the other.

Invariant natural killer T (iNKT) cells, the extensively studied innate-like T cells with an effector-memory phenotype (6), express an invariant TCR TRAV10-TRAJ18 chain in humans (TRAV11-TRAJ18 in mice and TRAV10-TRAJ18 in pigs) with a CDR3 of a constant length (7–10), and recognize self-lipids or microbe-derived lipids presented by the non-polymorphic MHC-Ib molecule, CD1d (11, 12). Besides existing in humans and mice, iNKT cells have also been described in pigs, which have similar properties to human and mouse iNKT cells, making pigs a useful animal model to study the function of iNKT cells (13, 14).

As the “cousins” of iNKT cells, MAIT cells also have received attention because of their high frequency in humans and their potential roles in disease. MAIT cells are a relatively recently described subset of innate-like T cells that were first reported in 1993 (15), and then termed MAIT cells in 2003 because of their semi-invariant TCR usage and their preferential location in mucosal tissues (16). Presently, MAIT cells are found in many tissues, and are known to be more abundant in some peripheral non-lymphoid and -mucosal tissues in humans and mice, such as liver and lung (17–19). Interestingly, the frequency of MAIT cells is much higher in humans than in mice (19, 20). MAIT cells express an evolutionarily conserved invariant TCRα chain (TRAV1-2-TRAJ33 in humans and TRAV1-TRAJ33 in mice) with a highly conserved CDR3α (CAVKDSNYQLIW in humans and CAVRDSNYQLIW in mice), which is paired with TCR Vβ chains with limited diversity (predominantly TRBV6 or TRBV20 in humans and TRBV13 or TRBV19 in mice) (15, 16, 21–24). There are high similarities in the MAIT TCR TRAV1-TRAJ33 chains among mammals (21), especially in TRAJ33 segments (>91%). Moreover, some MAIT cells have been observed that also express the non-canonical TCRα chains, with TRAJ12/20 usage in humans or with a variable CDR3α (16, 21, 22, 24).

Mucosal-associated invariant T (MAIT) cells are CD3^+^, and can also be classified into one of the three classical T cell phenotypes, CD4^+^CD8^−^, CD8^+^CD4^−^, or CD4^−^CD8^−^; the frequency and distribution of the three phenotypes among MAIT cells vary by tissue (4, 18, 23). MAIT cells express cytokine and chemokine receptors, such as IL-18Rα, IL-12Rβ, IL-7Rα, CCR9, CCR5, and CXCR6, which are characteristic of cytokine-dependent activation and the ability to traffic to mucosal tissues, respectively (4, 23, 25). MAIT cells also express transcription factors, including the promyelocytic leukemia zinc finger (PLZF) protein, T-bet, and the retinoic acid receptor (RAR)-related orphan receptor γ (RORγt, RORC), and have the capacity to secret IFN-γ and IL-17A, consistent with their effector phenotype (6, 23, 26–28). MAIT cells recognize vitamin B metabolites in the context of the highly phylogenetically conserved non-polymorphic MHC-related protein 1 (MR1) (16, 29, 30). It has been reported that genes encoding the two evolutionarily conserved proteins, the invariant TCRα chain and MR1, coevolved in mammals (31), implying an important role for MAIT cells in the host immune response that has been evolutionarily maintained. Indeed, it has been reported that MAIT cells are likely implicated in host defenses to both infectious and non-infectious diseases, such as tuberculosis, typhoid fever, influenza, multiple sclerosis, and colon cancer (32–35). To date, MAIT cells have been described in several mammals, including humans, mice, cows, sheep, and several non-human primates (16, 21, 36, 37), but not in pigs.

The fundamental immunological mechanisms between humans and pigs are very similar, so much so that pigs have been used as a preclinical animal model for human infectious diseases and vaccine development (38, 39). For the most part, all of the immune effector cells and molecules identified in humans also exist in pigs (40–42), including iNKT cells (13, 14). Moreover, our previous study demonstrated that pigs express the MR1 molecule (43). These observations imply that MAIT cells are present in pigs. Here, using porcine peripheral blood cells, we cloned the porcine homolog of the human MAIT cell TCRα chain, and then analyzed the TCRβ usage of porcine MAIT (pMAIT) expressing the TRAV1-TRAJ33 chain. We also analyzed the expression of TRAV1-TRAJ33 transcripts in several tissues and in three T-cell subpopulations (CD4^+^, CD8^+^, and CD4^−^CD8^−^). Finally, we examined the cell surface markers and transcription factors expressed by pMAIT cells expressing the TRAV1-TRAJ33 chain. Our study demonstrated that MAIT cells are present in pigs and have similar phenotypes to human and mouse MAIT cells.

## Materials and Methods

### Ethics Statement

All animal protocols were reviewed and approved by the Animal Administration and Ethics Committee of Lanzhou Veterinary Research Institute, Chinese Academy of Agricultural Sciences (Permit No. LVRIAEC-2009-006). The study was performed in strict compliance with the recommendations set forth in the Animal Ethics Procedures and Guidelines of the People's Republic of China. All efforts were made to minimize animal suffering and to reduce the numbers of animals used in the experiments.

### Animals, Tissues, and Cells

Three 5-month-old castrated male pigs (50% Duroc × 25% Landrace × 25% Large White) (P1, P2, and P3) that have been screened by RT-PCR and/or ELISA methods to rule out inapparent/potential infections are purchased from a local pig farm. RNA was isolated from peripheral blood, spleen, thymus, mesenteric lymph node (MLN), kidney, small intestine (SI), large intestine (LI), liver, and lung.

Peripheral blood mononuclear cells (PBMCs) were harvested from blood using Pig Lymphocyte Separation Medium (DAKEWE, China), according to the manufacturer's instructions. The preparations of single cell suspensions from spleen, liver, lung, and kidney were similar to our previous methods (43), and the single cell suspensions of SI were prepared according to the methods used by Weigmann et al. (44). The resultant cell suspensions were stored in liquid nitrogen until flow cytometry.

### Sequence of the MAIT TCRα Chain

The complete coding sequence (CDS) of the pMAIT cell TCRα chain was obtained by reverse transcription-PCR (RT-PCR) and 5′-rapid amplification of cDNA ends (5′-RACE). RNA was extracted from PBMCs, and then was reverse-transcribed into cDNA. Due to the evolutionarily conserved nature of the MAIT TCRα chain in mammals (21), the primers for amplification of pMAIT TCRα sequences were designed according to the pMAIT TCRα sequence predicted through the BLAT search in Ensemble genome browser using the human MAIT invariant TCRα sequence (accession number: HE862271.1), and were as follows: VF, 5′-GCACTGTGGGAGGAGGCATTGAG-3′; JR, 5′-TGGCTTTATAATTAGCTTGGTTCC-3′; and CR1, 5′-ACCACAGCCGCAGTGTCAT-3′ (Figure 2A). The reactions for PCR were as follows: denaturation at 94°C for 2 min, 35 cycles of denaturation at 94°C for 30 s, annealing at 58°C for 40 s and extension at 72°C for 1 min, and then extension at 72°C for 10 min with Ex Taq® HS (TaKaRa, China). 5′-RACE was used to amplify the 5′-ends of the TCRα sequence according to the SMARTer® RACE 5′/3′ Kit (TaKaRa, China) User Manual. The specific primers for 5′-RACE were 5′- GATTACGCCAAGCTTTAGCTTGGTTCCAGAGCCCC-3′ (outer primer) and 5′- GATTACGCCAAGCTTGACCTCTCCTCTCCCAACCA-3′ (inner primer) (Figure 2A). The PCR products were extracted from bands in 1.5% agarose gels and then sequenced.

### Flow Cytometry Sorting

CD4^+^, CD8^+^, and double negative (CD4^−^CD8^−^, DN) T cell populations were sorted from PBMCs and single cell suspensions of spleen, liver, lung, kidney, and SI using the BD Aria II flow cytometry (BD Bioscience, USA) (Figure 4A). Briefly, the cryopreserved cells were thawed, washed twice in warmed RPMI 1640 (Gibco, USA) supplemented with 2% fetal calf serum (FCS, Gibco, USA), and then resuspended in 100 μl sorting buffer (PBS containing 2% FCS and 200 U/ml RNAsin, Promega, USA). Cells were stained with FITC-conjugated anti-pig CD3 (1:200, clone BB23-8E6-8C8, BD, USA), PE-Cy7-conjugated anti-pig CD4 (1:200, clone 74-12-4, BD, USA), Alexa Fluor 647-conjugated anti-pig CD8 (1:200, clone 76-2-11, BD, USA), and PE-conjugated anti-pig γδ TCR (1:200, clone MAC320, BD, USA) for 30 min at 4°C in the dark. After washing twice with sorting buffer, cells were incubated with 7-AAD (1:40, BD, USA) for 10 min at 4°C in the dark. Single cell suspensions were prepared using a 40-μm cell strainer, and then sorted immediately. 10,000 to 50,000 cells were collected for each target cell population with >90% purity. The obtained T cell populations were used to extract RNA for qPCR analysis.

### Quantitative PCR for TRAV1-TRAJ33 Transcript Expression in Tissues and Cell Populations

The frequency of TRAV1-TRAJ33 transcripts in tissues and cell populations was analyzed using quantitative PCR (qPCR). Total RNA was isolated from tissues and sorted T cell populations using TRIzol® Reagent (Invitrogen, USA) and RNeasy Mini Kit (Qiagen, Germany), respectively, followed by cDNA synthesis using PrimeScript^TM^ RT reagent Kit with gDNA Eraser (TaKaRa, China). In this experiment, a construct containing the pMAIT TCR Vα-Jα-Cα sequence was used as a standard to quantify the expression of Vα-Jα and Cα in each sample. The frequency of TRAV1-TRAJ33 transcripts in the total TCRα cDNA was determined by taking the ratio of (TRAV1-TRAJ33)/TRAC (21, 36).

The primers targeting the TRAV1-TRAJ33 and TRAC were 5′-TCATACCTTAGTCACTCTGTTGC-3′ (VJF), 5′-TCCAGAGCCCCAGATCAACT-3′ (VJR), 5′-TCAGCTTGAGCAGGACTGTG-3′ (CF), and 5′-TGCTGGAAGGTGCTTTGACA-3′ (CR2) (Figure 2A). qPCR was performed in a 20-μl reaction volume containing cDNA and 150 nM of each primer in SYBR® Premix Ex Taq^TM^ II (TaKaRa, China). The reaction conditions consisted of 30 s at 95°C followed by 39 cycles of 95°C for 5 s and 60°C for 30 s and were performed using the CFX96 Real-Time Systems (Bio-Rad, USA).

### Single-Cell PCR Analysis of the TCRβ Usage of MAIT Cells Expressing the TRAV1-TRAJ33 Chain

**Figure 1:**
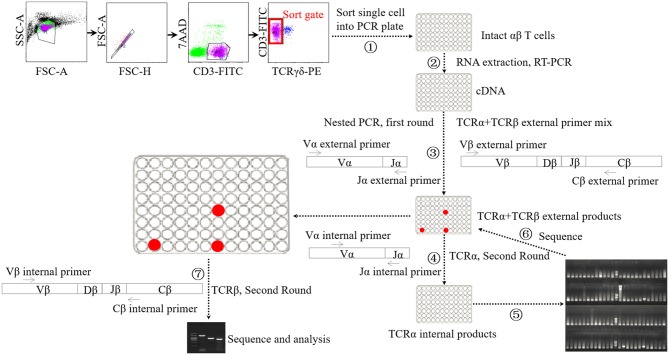
Schematic diagram of single-cell PCR assay. ① Sorting single αβ T cell into 96-well PCR plate. ② Performing cDNA synthesis on individual cells in 96-well plate without RNA extraction step. ③ Performing the first-round nested PCR with external primers targeting α chain and β chains using resultant cDNA. ④ Performing the second-round PCR with internal primers targeting α chain using the products of first-round nested PCR. ⑤ Electrophoresis analysis of the products of forth step. ⑥ Sequencing analysis of the products of suspected positive TRAV1-TRAJ33 cell. ⑦ Performing the second-round PCR with internal primers targeting β chains using the products of TRAV1-TRAJ33^+^ cells.

Single-cell PCR analysis was performed according to the methods used previously (Figure 1) (45–47). Briefly, cellular staining was conducted with the methods described above. Upon staining with fluorochrome-conjugated monoclonal antibodies and 7-AAD, single lung αβ T cells were sorted into a 96-well PCR plate with a full skirt (Brand, Germany), containing 2.5-μl reverse transcription reaction mixes or not, using BD Aria II flow cytometry. After sorting, the plates loaded with single cells were sealed using plate sealer film (Applied Biosystems, USA), centrifuged, and frozen at −80°C until use.

cDNA synthesis was conducted on the single cells using the iScript cDNA Synthesis Kit (Bio-Rad, USA) without RNA extraction step, using 2.5-μl reaction mixes composed of 0.5 μl 5 × iScript reaction mix, 0.5 μL iScript reverse transcriptase, 0.25 μl 1% Triton X-100 (Life Technologies, USA), and 1.25 μl nuclease-free water. The reaction conditions consisted of 5 min at 25°C followed by 45°C for 45 min and 85°C for 5 min. Each 2.5-μl cDNA product was then subjected to two rounds of nested PCR using a Taq DNA polymerase-based PCR kit (Qiagen, Germany) in a 25-μl reaction mix. The first round of multiplex PCR was performed with 1 U Taq DNA polymerase, 2.5 μl 10 × PCR buffer, 0.5 μl 10 mM dNTP, 0.5 μl external primer mixture of TRAV1 forward and TRAJ33 reverse along with 21 TRBV forward oligonucleotides (each 2.5 pmol), and 0.5 μl single TRBC reverse primer (10 pmol). The PCR conditions consisted of 5 min at 95°C, followed by 35 cycles of 95°C for 20 s, 52°C for 20 s and 72°C for 1 min, and then extension at 72°C for 10 min. Then the first-round PCR products were subjected to the second-round PCR using EX Taq DNA polymerase (TaKaRa, China) with the internal primer mixture of TRAV1 forward primer and TRAJ33 reverse primer (each 5 pmol) to identify T cells expressing the TRAV1-TRAJ33 transcript with a canonical CDR3α segment (MAIT cells). The second-round PCR was also performed to analyze TCRβ usage using the first-round PCR products of positive cells and the internal primer mixture of 21 TRBV forward primers and TRBC reverse primer. All the primers are listed in Table S1. The PCR products were then purified and sequenced.

### Single-Cell PCR Analysis of the Expression of Cell Surface Markers and Transcription Factors by MAIT Cells

To examine the cell surface markers and transcription factors expression by pMAIT cells expressing the TRAV1-TRAJ33 chain, single αβ T cells were sorted from PBMC, and then were used to detect the expression of IL-18Rα, IL-12Rβ, IL-7Rα, CCR9, CCR5, and CXCR6, or PLZF, T-bet, and RORC. The strategy for the single-cell analysis was the same as described above. The primers targeting these markers and transcription factors are listed in Table S2.

### Statistical Analysis

Statistical analysis was performed using SPSS 13.0 software (SPSS Inc., Chicago, USA). Non-parametric Mann-Whitney *U*-tests were used to analyze the data of qPCR.

## Results

### Detection and Characterization of the Porcine TRAV1-TRAJ33 Chain

**Figure 2:**
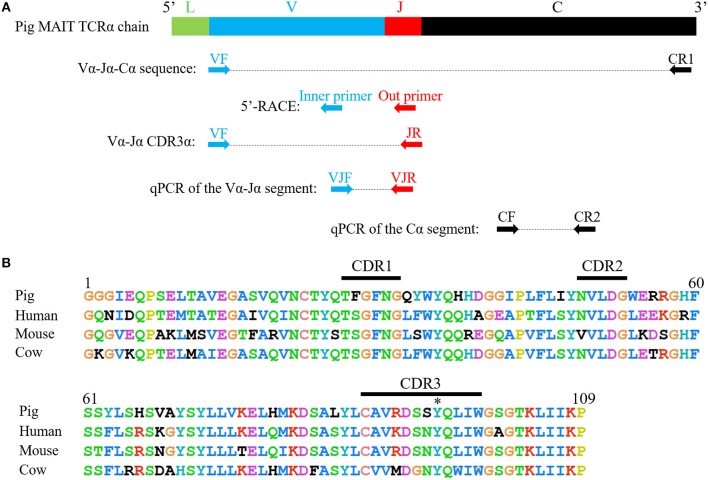
Cloning and characterization of the porcine MAIT TCR Vα-Jα chain. **(A)** A scheme showing the position of the primers in porcine MAIT TCRα sequence. L, Leader sequence; V, TRAV1 gene; J, TRAJ33 gene; C, Constant gene. **(B)** Alignment of amino acid sequences of pig, human, mouse, and cow MAIT TCR Vα-Jα chain. CDR1, CDR2, and CDR3 segments are highlighted with black line. Y95 is highlighted with *.

Because of the highly conserved nature of the MAIT TCRα chain among mammalian species, the porcine orthologous segments of the MAIT TCR Vα segment (TRAV1-2 in humans and TRAV1 in mice) and the Jα segment (TRAJ33 in humans and mice) were predicted using the human MAIT TRAV1-2 and TRAJ33 sequences, and then named TRAV1 and TRAJ33, respectively, according to the annotation approach used by Butler et al. (48), Yamamoto et al. (49), and Uenishi et al. (50). Porcine TRAV1 and TRAJ33 segments are located on chromosome 7 in the pig genome. Porcine T cells that express the homologous TCRα chains were identified with RT-PCR from PBMC cDNA from P1, P2, and P3, and the complete CDS of the TCRα chain (accession number: MN086839) was obtained with 5′-RACE to amplify the 5′-end of the TCRα sequence. TRAV1-TRAJ33 rearrangements with a canonical CDR3α (CAVRDSSYQLIW) were obtained by sequencing several clones from the RT-PCR products of each pig. The amino acid sequence of the porcine TRAV1-TRAJ33 (pTRAV1-TRAJ33) has a high degree of similarity with the human, mouse, and cow MAIT invariant TCRα chains, especially in the complementarity-determining regions (CDRs) (Figure 2B). A critical tyrosine residue, Y95, which has been shown to be required for MAIT cell activation (51), is also present in the porcine CDR3α. Furthermore, several TCRα chain sequences comprised of TRAV1 joined to different TRAJ segments were also obtained (data not shown).

To analyze the CDR3α diversity in the pTRAV1-TRAJ33 transcripts, primers VF and JR, specific for the TRAV1 and TRAJ33 segments, respectively, were used to amplify the TCR Vα-Jα products from PBMC cDNA of P1, P2, and P3. As shown in Table 1, a pTRAV1-TRAJ33 transcript with a canonical CDR3α, corresponding to the invariant chain, accounted for the most of the sequences obtained from each pig, and 71% of the pTRAV1-TRAJ33 transcripts from the three pigs encoded the invariant TCRα chain, indicating a strong selection for this canonical transcript. This transcript displayed a highly conserved CDR3α sequence with the same length in the human, mouse, and cow. Other pTRAV1-TRAJ33 transcripts with different CDR3α sequences (11%), and some out-of-frame sequences (18%) were also obtained from these PCR products. Taken together, these results suggest that porcine T cells express the orthologous TCR Vα-Jα transcripts of the human or mouse MAIT invariant TCRα chain, indicating MAIT cells are present in pigs, and these transcripts contain a conserved canonical CDR3α sequence, although some non-canonical sequences were also observed.

**Table 1:** Sequences of TRAV1-TRAJ33 transcripts from three pigs.

**Animal**	**CDR3α**	**Number**
P1	CAV RDSSYQ LIW	15
	CAV GGSSYQ LIW	1
	CAV NGSSYQ LIW	1
	Out of frame	3
P2	CAV RDSSYQ LIW	14
	CAVRDQPLDSSYQ LIW	1
	Out of frame	3
P3	CAV RDSSYQ LIW	10
	CAV RVDSSYQ LIW	1
	CAV RDPVDSSYQ LIW	1
	CAV RPMDSSYQ LIW	1
	Out of frame	4
Total	Canonical sequence	39 (71%)
	Same CDR3α length, different sequence	2 (4%)
	Different CDR3α length	4 (7%)
	Out of frame	10 (18%)

### TCRβ Chain Repertoire of Porcine MAIT Cells Expressing the TRAV1-TRAJ33 Chain

Human or mouse MAIT cells bearing a canonical invariant TCRα chain utilize a limited diversity of the TCRβ repertoire (21). In order to estimate the TCRβ chain repertoire of pMAIT cells, a single-cell PCR assay was employed. Forty-one pMAIT cells were obtained from three pigs, and their respective TCRβ transcripts were sequenced. As shown in Table 2, TCRβ chains expressed by TRAV1-TRAJ33^+^ cells were heterogeneous, and 14 different Vβ segments were obtained. As expected, the use of Vβ segments was biased, and was dominated by TRBV20S (9/41) and TRBV29S (11/41), consistent with the TCR Vβ usage by human or mouse MAIT cells (21). Furthermore, we also analyzed the usage of Jβ and CDR3β segments. There was no apparent restriction in the Jβ usage, consistent with human or mouse MAIT TCR Jβ usage (21); the number of amino acids in the CDR3β ranged from 8 to 16. Interestingly, TRBV29S was more inclined to join to the Jβ2.4 segment, and the CDR3β sequences of TRBV29S-Jβ2.4 were highly conserved, only two of which had a single amino acid change at position 7. Taken together, these results suggest that the use of TCRβ chain of pMAIT cells expressing the TRAV1-TRAJ33 chain is more diverse than TCRα chain, and the Vβ segments used are predominantly TRBV20S and TRBV29S.

**Table 2:** TCRβ sequences of porcine TRAV1-TRAJ33^+^ cells.

**TRAV-TRAJ**	**TRBV**	**CDR3β**	**TRBJ**	**Number**
TRAV1-TRAJ33	TRBV29S	SAGYIRGAGGNTQHF	Jβ2.4	8
TRAV1-TRAJ33	TRBV29S	SAGYIRVAGGNTQHF	Jβ2.4	2
TRAV1-TRAJ33	TRBV29S	SAGDWVPLYSETQYF	Jβ2.5	1
TRAV1-TRAJ33	TRBV20S	ATSYSQTQYF	Jβ2.5	1
TRAV1-TRAJ33	TRBV20S	GAKRALTEGNTQHF	Jβ2.4	2
TRAV1-TRAJ33	TRBV20S	GASVWGGDTEVFF	Jβ1.1	2
TRAV1-TRAJ33	TRBV20S	GASNSRGAGTDPLYF	Jβ2.3	1
TRAV1-TRAJ33	TRBV20S	ALGQGRADPLYF	Jβ2.3	1
TRAV1-TRAJ33	TRBV20S	ASGRRGSETQYF	Jβ2.5	1
TRAV1-TRAJ33	TRBV20S	ARQRGTGLSGYAEQHF	Jβ2.1	1
TRAV1-TRAJ33	TRBVX	FEDNYAEQHF	Jβ2.1	1
TRAV1-TRAJ33	TRBVX	SEHGGAAQLYF	Jβ2.2	2
TRAV1-TRAJ33	TRBVX	SDWIGKSLGSTQHF	Jβ2.4	1
TRAV1-TRAJ33	TRBVX	SEDSWGSPTAEQHF	Jβ2.1	1
TRAV1-TRAJ33	TRBV7S	RQGLTDPLYF	Jβ2.3	2
TRAV1-TRAJ33	TRBV7S	LRWRDLNYNSPLHF	Jβ1.6	1
TRAV1-TRAJ33	TRBV4S	RGRGGYDYNF	Jβ1.2	1
TRAV1-TRAJ33	TRBV4S	PAGGIAGSPLHF	Jβ1.6	1
TRAV1-TRAJ33	TRBV5S	LDAMGQTQYF	Jβ2.5	1
TRAV1-TRAJ33	TRBV5S	PAFGQSRYPLYF	Jβ2.3	1
TRAV1-TRAJ33	TRBV11S	SRRDTNEKLIF	Jβ1.4	1
TRAV1-TRAJ33	TRBV11S	QHPRGGGSPSYEQIF	Jβ2.7	1
TRAV1-TRAJ33	TRBV10S	MGGQTQYF	Jβ2.5	1
TRAV1-TRAJ33	TRBV12S	ETVNRNTGQLYF	Jβ2.2	1
TRAV1-TRAJ33	TRBV15S	RDRAMGYSQTQYF	Jβ2.5	1
TRAV1-TRAJ33	TRBV19S	PWLRGASQNTQHF	Jβ2.4	1
TRAV1-TRAJ33	TRBV25S	GDRGENTQHF	Jβ2.4	1
TRAV1-TRAJ33	TRBV27S	VSDRGITDPLYF	Jβ2.3	1
TRAV1-TRAJ33	TRBV30S	WVGADSYEQIF	Jβ2.7	1

*αβ T cells from porcine lung were sorted as single cells into 96-well plate, and then nested PCR was performed to identify TRAV1-TRAJ33^+^ cells, followed by amplifying TCRβ sequences from these TRAV1-TRAJ33^+^ cells. The named principle of Vβ and Jβ genes was according to the nomenclature used by Butler et al. (48). CDR3β was defined in accordance with Kabat et al. (52)*.

### Relative Abundance of Porcine MAIT Cells in Tissues

**Figure 3:**
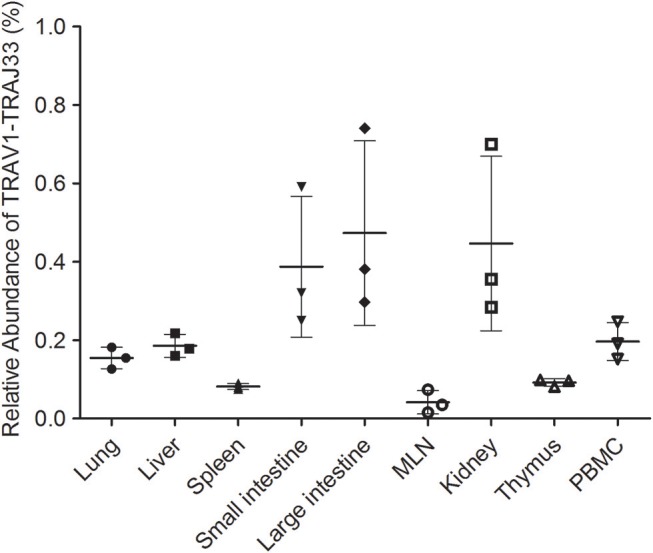
Relative abundance of pMAIT TCRα in PBMC and tissues. Two qPCR amplifications were performed with each cDNA sample prepared from PBMC and tissues to analyze the expression of MAIT TCR Vα-Jα and total TCRα (Cα). The relatively abundance of MAIT TCRα mRNA in total TCRα cDNA then was determined through taking the ratio of Vα-Jα/Cα. Samples are taken from three pigs, and data are representative of three experiments on the same samples from each pig. Bars indicate mean ± SD. MLN, mesenteric lymph node; PBMC, peripheral blood mononuclear cell.

Due to the lack of an anti-pTRAV1 antibody or a porcine MR1-ligand tetramer, we relied on the qPCR of the TRAV1-TRAJ33 TCRα chain to track MAIT cells (18, 21, 36). Peripheral blood and several tissues, collected from P1, P2, and P3, were used to extract RNA products, which were then subjected to two qPCR amplifications to assess the expression of TRAV1-TRAJ33 and total TCRα in each sample. We quantified the amounts of TRAV1-TRAJ33 transcripts and Cα transcripts, and then compared that to the amount of Cα transcripts to determine the relative abundance of MAIT TCR Vα-Jα in total TCRα cDNA. The qPCR efficiencies of this experiment were between 95 and 105%. As shown in Figure 3, the abundance of pTRAV1-TRAJ33 tended to be different, although there were no statistical significant differences between peripheral non-lymphoid tissues and lymphoid tissues. The expression of TRAV1-TRAJ33 transcripts was detected in the LI (mean 0.47%), the kidney (mean 0.45%), the SI (mean 0.39%), PBMC (mean 0.2%), the liver (mean 0.19%), the lung (mean 0.16%), the thymus (mean 0.09%), the spleen (mean 0.08%), and the MLN (mean 0.04%). These results suggest that pMAIT cells expressing the TRAV1-TRAJ33 chain are present in all tested tissues.

### T Cell Subsets of Porcine MAIT Cells in Tissues

**Figure 4:**
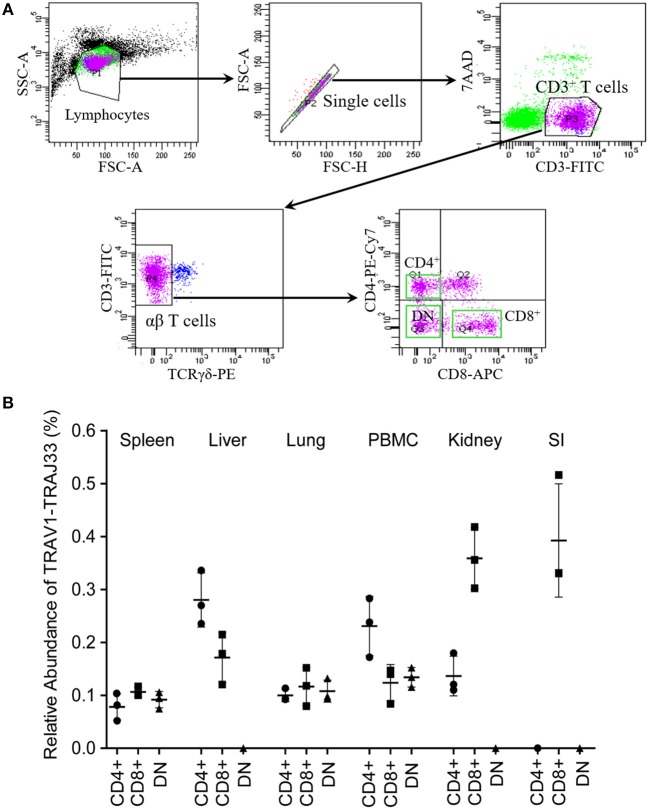
T cell subsets of porcine MAIT cells in tissues. **(A)** FACS profiles for sorting CD4^+^, CD8^+^, and DN (double negative, CD4^−^CD8^−^) T cell subsets from single cell suspensions of porcine spleen. **(B)** Relative abundance of pMAIT TCRα in three T-cell subpopulations from peripheral blood and tissues. CD4^+^, CD8^+^, and DN T cells were sorted from different samples using flow cytometry, and then were used to synthesize cDNA for qPCR analysis. Two qPCR amplifications were performed with each cDNA sample prepared from tissues to analyze the expression of MAIT TCR Vα-Jα and total TCRα (Cα). The relatively abundance of MAIT TCRα mRNA in total TCRα cDNA then was estimated through taking the ratio of Vα-Jα/Cα. Samples are taken from three pigs, and data are representative of three experiments on the same samples from each pig. Bars indicate mean ± SD. PBMC, peripheral blood mononuclear cell; SI, small intestine.

Mucosal-associated invariant T (MAIT) cells exist in three phenotypically distinct T cell subpopulations in humans and mice, CD4^+^CD8^−^, CD4^−^CD8^+^, and CD4^−^CD8^−^ (22), and their frequency varies in different tissues in mice (18, 23). To investigate the frequency of CD4^+^, CD8^+^, and DN pMAIT cells in different tissues, we used the same strategy described above. Single cell suspensions were prepared from several tissues, and CD4^+^, CD8^+^, and CD4^−^CD8^−^ T cells were obtained from these suspensions using flow cytometry sorting. The qPCR efficiencies of this experiment were between 90 and 105%. As shown in Figure 4B, TRAV1-TRAJ33 transcripts could be expressed by CD4^+^, CD8^+^, and DN T cells. However, the frequencies of each subsets of MAIT cells in the various tissues tended to be different. In PBMC, spleen, and lung, CD4^+^, CD8^+^, and DN T cells expressed TRAV1-TRAJ33 transcripts. In the liver, kidney, and SI, there was no detectable expression of TRAV1-TRAJ33 transcripts in DN T cells. Furthermore, we did not detect TRAV1-TRAJ33 expression in CD4^+^ T cells of the SI. TRAV1-TRAJ33 transcripts were expressed by CD8^+^ T cells in all tested tissues. Collectively, MAIT cells can express CD4, CD8 or neither, and the frequency of three subsets varies in different tissues, similar to their counterparts in mice (18, 23).

### Cell Surface Marker Expression on Porcine MAIT Cells

Besides the CD4 or CD8 coreceptor, human and mouse MAIT cells also express cytokine and chemokine receptors, such as IL-18Rα, IL-12Rβ, IL-7Rα, CCR9, CCR5, and CXCR6 (4, 23). To examine the cell surface markers on pMAIT cells, a single-cell PCR assay was performed with the primers targeting IL-18Rα, IL-12Rβ, IL-7Rα, CCR9, CCR5, and CXCR6 using the same methods described above. As shown in Table 3, we obtained 19 pMAIT cells from three pigs, and found that these cells could express the transcript of IL-18Rα, IL-7Rα, CCR9, CCR5, and/or CXCR6, just as their human and mouse counterparts do, implying that pMAIT cells can be activated in a cytokine-mediated manner and have the ability to migrate to mucosal tissues. However, not all cells expressed all of these markers, with one exception, IL-18Rα, which was expressed in all cells. Moreover, IL-12Rβ transcript expression was not detected in any cell. Collectively, pMAIT cells bearing the TRAV1-TRAJ33 chain express cytokine and chemokine receptors in a manner similar, but not identical, to human and mouse MAIT cells.

**Table 3:** The expression of cell surface markers on pMAIT cells.

**Pig**	**Cell**	**TRAV-TRAJ**	**IL-18Rα**	**IL-12Rβ**	**IL-7Rα**	**CCR9**	**CCR5**	**CXCR6**
P1	1F9^**#**^	TRAV1-TRAJ33	+	–	+	+	–	–
	3E12	TRAV1-TRAJ33	+	–	–	+	+	+
	4H2	TRAV1-TRAJ33	+	–	+	+	–	+
	7A5	TRAV1-TRAJ33	+	–	–	+	–	–
	9C8	TRAV1-TRAJ33	+	–	–	+	–	+
	11A10	TRAV1-TRAJ33	+	–	+	–	–	+
	12G1	TRAV1-TRAJ33	+	–	+	–	+	+
	16A5	TRAV1-TRAJ33	+	–	+	–	+	+
P2	18A10	TRAV1-TRAJ33	+	–	–	+	–	+
	23B8	TRAV1-TRAJ33	+	–	–	+	–	+
	25D8	TRAV1-TRAJ33	+	–	–	+	–	–
	26E11	TRAV1-TRAJ33	+	–	+	–	+	–
	28F11	TRAV1-TRAJ33	+	–	+	–	–	+
P3	29G1	TRAV1-TRAJ33	+	–	+	+	–	+
	31H2	TRAV1-TRAJ33	+	–	–	+	–	+
	34B5	TRAV1-TRAJ33	+	–	–	–	+	–
	35C7	TRAV1-TRAJ33	+	–	–	+	+	–
	36B10	TRAV1-TRAJ33	+	–	+	–	–	+
	39D3	TRAV1-TRAJ33	+	–	+	+	–	+

# *“1” is the number of the 96-well PCR plate; “F9” is the well number in this plate*.

### Transcription Factor Expression by Porcine MAIT Cells

Human and mouse MAIT cells are known to express transcription factors PLZF and T-bet and/or RORC, which endows them effector phenotype and regulates the ability to produce IFN-γ and/or IL-17A (6, 17, 23, 26, 53, 54). To investigate whether these transcription factors are also expressed by pMAIT cells, the single-cell PCR assay was employed again to detect the transcript expression of PLZF, T-bet, and RORC by pMAIT cells from peripheral blood. We obtained 11 pMAIT cells, and found that these cells can express PLZF (10/11), T-bet (8/11) and RORC (2/11) (Table 4), as their human and mouse counterparts do, indicating that pMAIT cells bearing the TRAV1-TRAJ33 chain express the transcription factors known to regulate the effector phenotype, and may also display a Th1 and/or Th17 pattern of cytokine secretion.

**Table 4:** The expression of transcription factors by pMAIT cells.

**Pig**	**Cell**	**TRAV-TRAJ**	**PLZF**	**T-bet**	**RORC**
P1	1D9^*^	TRAV1-TRAJ33	+	+	–
	2E10	TRAV1-TRAJ33	+	+	–
	5F10	TRAV1-TRAJ33	+	+	–
	7C6	TRAV1-TRAJ33	+	–	–
	8A3	TRAV1-TRAJ33	+	+	+
	12C7	TRAV1-TRAJ33	–	+	–
P2	14G5	TRAV1-TRAJ33	+	+	–
	17F5	TRAV1-TRAJ33	+	+	–
	18G10	TRAV1-TRAJ33	+	–	+
P3	20G12	TRAV1-TRAJ33	+	+	–
	23A10	TRAV1-TRAJ33	+	–	–

* *“1” is the number of the 96-well PCR plate; “D9” is the well number in this plate*.

## Discussion

Mucosal-associated invariant T (MAIT) cells have been receiving extensive attention in the fields of immunology research and clinical practice because of their special antigenic repertoire and their potential role in immunotherapy. MAIT cells expressing an invariant TCRα chain (TRAV1-2-TRAJ33 in humans and TRAV1-TRAJ33 in mice) have coevolved with their restricting molecule, MR1, in mammals (31). This MR1-MAIT cell axis has been delineated in humans, mice, cows, and other animals (21, 31, 36, 37, 55). Our previous studies showed that MR1 is expressed in multiple pig tissues and cells (43), implying that MAIT cells are present in pigs. In this study, we demonstrated the presence of MAIT cells expressing a homologous TRAV1-TRAJ33 chain paired with a limited diversity of Vβ segments (predominantly TRBV20S or TRBV29S) in pigs and found that these cells have similar phenotypes to human and mouse MAIT cells.

Human, mouse, cow, and sheep MAIT cells express a highly conserved canonical TCRα chain containing a nearly identical CDR3α segment (21, 36). Our results showed that pigs also express a canonical TCRα transcript (TRAV1-TRAJ33) that is orthologous to human MAIT TCRα chain (TRAV1-2-TRAJ33) and has a high sequence similarity to the MAIT TCRα chain of other species; therefore, we inferred that this transcript could represent the pMAIT TCRα chain. This TRAV1-TRAJ33 transcript contains a canonical CDR3α segment (CAVRDSSYQLIW) that is more similar to the mouse MAIT CDR3α segment (CAVRDSNYQLIW) than it is to the human MAIT CDR3α segment (CAVKDSNYQLIW). Interestingly, the porcine CDR3α (CAVRDSSYQLIW) seems to be a combination of the CDR3α sequences of human MAIT TRAV1-2-TRAJ20 (CAVRDGDYKLSF), TRAV1-2-TRAJ12 (CAVMDSSYKLIF), and TRAV1-2-TRAJ33 (CAVKDSNYQLIW). More importantly, the Y95 residue that is crucial for MAIT cell activation is also conserved in the pMAIT TCRα chain (51). It is well-accepted that CDR3α is the key determinant of specificity in antigen recognition (56). Therefore, these results suggest that pMAIT cells may have the same antigenic repertoire as human and mouse MAIT cells. Besides the canonical CDR3α segment, we also obtained some non-canonical CDR3α segments with sequences of different length, same length but different sequences, or out-of-frame sequences. Similar observations have also been reported in human, mouse, and cow MAIT cells (16, 21, 36).

Because of the limited diversity of TCR Vβ usage of human and mouse MAIT cells (21–23), we analyzed the TCRβ chain repertoire of pMAIT cells expressing the TRAV1-TRAJ33 chain using the single-cell PCR assay (45–47). Our results showed that the repertoire of Vβ segments used by pMAIT cells was diverse but biased toward TRBV20S (9/41) and TRBV29S (11/41); pTRAV1-TRAJ33 was also paired with TRBVX, TRBV7S, TRBV4S, TRBV5S, TRBV11S, TRBV10S, TRBV12S, TRBV15S, TRBV19S, TRBV25S, TRBV27S, and TRBV30S. Interestingly, human TRBV20 which has 73% similarity to TRBV20S is also predominantly used by human MAIT cells (21), indicating a similarity of TCRβ usage between human and porcine MAIT cells. Furthermore, although pMAIT cells have a biased usage of TCR Vβ segments, there is no obvious restrictions in Jβ usage, with one exception: TRBV29S prefers joining to Jβ2.4 with a highly conserved CDR3β, which is consistent with another published report (48). pMAIT cells also display a diverse CDR3β usage. Moreover, the same TCRα and β chain with the same sequence was used by different cells from the same pig (data not shown), indicating oligoclonal expansions of these subsets *in vivo*, which is consistent with MAIT cell features (17, 21).

Mucosal-associated invariant T (MAIT) cells are abundant in humans, but less frequent in mice (17, 21–23). In our study, qPCR was performed using two primer sets, VJF and VJR, and CF and CR2, which are specific for TRAV1-TRAJ33 and Cα, respectively. This strategy was used to analyze the abundance of pMAIT cells in tissues (15, 18, 21, 36), although the high expression of TRAV1-TRAJ33 transcripts may not always be equal to a large number of MAIT cells because of the possible disequilibrium expression of this transcripts by cells (21) and the mix of non-canonical TRAV1-TRAJ33 transcripts (16). The frequency of pMAIT cells was lower than that of MAIT cells in humans (19, 21); however, similar to mouse MAIT cells, MAIT cells were low in pigs, although TRAV1-TRAJ33 transcripts were expressed in all tissues. We also investigated the abundance of pMAIT cells in three T-cell subpopulations: CD4^+^, CD8^+^, and DN T cells. Our results showed that pMAIT cells also include CD4^+^, CD8^+^, and DN subsets but with varied frequencies in different tissues. The CD8^+^ subset is present in all tissues, the CD4^+^ subset is not present in SI, and the DN subset is not present in liver, kidney, and SI, suggesting different subset requirements in these tissues. It is worth noting that the sensitivity of qPCR using SYBR green dye may not be sufficient to detect very low levels of expression of the TRAV1-TRAJ33 transcripts in the specific subsets. Moreover, the three subsets of pMAIT cells we defined using CD4 and CD8 expression may or may not align with other species due to considerable variation in expression of these molecules between pigs vs. humans and mice.

Human and mouse MAIT cells express many of the same cytokine and chemokine receptors (17, 23). We found that, in line with their counterparts in human and mouse, pMAIT cells can also express cytokine receptors IL-18Rα and IL-7Rα, and chemokine receptors CCR9, CCR5, and CXCR6, indicating that their potential reactivity to cytokine stimulation (4, 57, 58) and their ability to traffic to the gut, liver, kidney, and lung (17, 59–63), respectively. Indeed, consistent with their expression of chemokine receptors, pMAIT cells are abundant in the intestine, liver, kidney, and lung. In our study, we did not detect the IL-12Rβ expression by 19 TRAV1-TRAJ33^+^ cells, although it is expressed by human and mouse MAIT cells. This may be because the sample size was small, but it cannot exclude the possibility that IL-12Rβ is not expressed by pMAIT cells from peripheral blood. Given that human and mouse MAIT cells express PLZF, T-bet and RORC, we hypothesized that pMAIT cells also express these transcription factors, and indeed, we found that pMAIT cells do express PLZF, T-bet and RORC. PLZF is recognized as a primary controller of innate-like T cell development, such as iNKT cells and MAIT cells (23, 27, 64, 65) and pMAIT cell development may be similar to other MAIT cells. Furthermore, recent studies showed that mouse MAIT cells can be divided into at least two functional subsets according to the differential expression of T-bet and RORC (23, 26); therefore, our data imply that there may also be at least two subsets of MAIT cells with different functions in pigs. It should be mentioned that in our approach, we did not compare to any other T cell subsets to confirm that these cytokine and chemokine receptors are enriched on TRAV1-TRAJ33^+^ cells.

In conclusion, we described a new member of the MAIT-cell family, pMAIT cells, which share many similarities with human and mouse MAIT cells. In view of the higher degree of similarity between the human and pig immune systems (>80%) (66), as compared to the similarity between human and mouse (<10%) (38), pigs may be an important intermediate model to evaluate the role of MAIT cells in disease. Future efforts will focus on the preparation of an anti-pTRAV1 antibody and MR1-ligand tetramer to better study the role of pMAIT cells in infectious and non-infectious diseases.

## Data Availability

The data (accession number: MN086839) generated in this study can be found in the GenBank sequence database (https://www.ncbi.nlm.nih.gov/genbank/), and the raw data supporting the conclusions of this study will be made available by the authors, without undue reservation, to any qualified researcher.

## Author Contributions

JC designed the outline, organized the text, and critically revised the manuscript. XX, XH, WW, and BJ prepared samples and performed experiments. XX, XM, and BL carried out data analysis and wrote the manuscript. KL and SY helped design the experiments. All authors reviewed and approved the final version of the manuscript.

### Conflict of Interest Statement

The authors declare that the research was conducted in the absence of any commercial or financial relationships that could be construed as a potential conflict of interest.
