# Supplementary material for: Mucosal-Associated Invariant T Cells Expressing the TRAV1-TRAJ33 Chain Are Present in Pigs
Source: Front Immunol. 2019 Sep 3;10:2070. doi: 10.3389/fimmu.2019.02070 (PMC6735250; doi:10.3389/fimmu.2019.02070)
Supplement: Supplementary file 1 [file Table_1.DOCX]

**TABLE S1 |** Primers targeting TRAV1-TRAJ33 and TRB genes.

| Genes | External primer sequence 5’->3’ | Internal primer sequence 5’->3’ |
| --- | --- | --- |
| TRAV1 | CTGTGGGAGGAGGCATTGAG | TCATACCTTAGTCACTCTGTTGC |
| TRAJ33 | TGGCTTTATAATTAGCTTGGTTCC | TCCAGAGCCCCAGATCAACT |
| TRBV2S | GGGAGTAGGCCACATGGAAC | TCGCCACCACATCACAAAGT |
| TRBV3S | GGTCCCTAAAATGTGAGCAAAAG | ACAGTTCCACGTCGCTTCTT |
| TRBV4S | CAGATACCTGGTCCTGGGAA | GGCTTGACTGCCCAGAGAG |
| TRBV5S | CACCGAGACATCTGATTAAAGC | ACCAAAAGCAAAGCGCCAAA |
| TRBV6S | ACTGAACCGTGCCCAAGA | CGGCTACAGCGTCTCAAGAT |
| TRBV7S | AGCTTTTGTCTCCACAGGTCA | GTAATGGCCGGTTCTCTGCT |
| TRBV9S | TCTGAGCTGAAATTGCTCTCC | CTCAGCCCTGTACCTCTGTG |
| TRBV10S | CCTGTGATGTTGGCATCCTT | CGAGACCAAGCATGGAGGAT |
| TRBV11S | TGTTTCTCAGTTGCCCCAGA | ATTTTGCAGAGAGGCCCGAA |
| TRBV12S | CACCCAGACACGAGGTGA | GTACAGACAGACCTTGGGGC |
| TRBV12-AS | CAACAACGGGTCTCCTGTG | ACAAGTCAGGGATGCCCAAG |
| TRBV15S | ACCCTTCTTTCTGTTCCCTTG | GACTCCGCCGTGTATCTCTG |
| TRBV19S | CCCAGGACAAGGACTGAGA | TCAGGCTACAATGCCTCTCG |
| TRBV20S | CCCCTGCTGATAGCAACTTC | TCTCATCAGCCACCCAAACC |
| TRBV21S | CGAGTGCCTCAAGAACTCCT | GGGAATCAATTCCACTGAGGCA |
| TRBV24S | CCCAGATCCCAAGGAACAAG | GGGCTCCGGTCCATCTACTA |
| TRBV25S | CGGGGTTAATTCCACAGAG | CACCAGCCCTTCACAGACAT |
| TRBV27S | TCAAGGAACGTTGATTTGGT | GCCGAATTTCCCCTTGATCC |
| TRBV29S | ACCGTCAGCTTCTAGGACAAAG | ATTAGCCGCCCAAACCTGAT |
| TRBV30S | TGACCAGAAAGATCCTGAAAAG | TCCAGAACTTCAACGCCTCC |
| TRBVXS | AGGCCACATCCCTTCCT | AGCAGATTATCAGGCCACGC |
| TRBC | ATCTCCGCTTCCGATGGT | TTGGGTGGTCTCACCTGCT |

Primers targeting TRAV1 and TRBV genes are forward. Primers targeting TRAJ33 and TRBC genes are reverse.

**TABLE S2 |** Forward and reverse primers targeting cell surface marker and transcription factor genes (both external and internal).

| genes | External forward primer 5’->3’ | Internal forward primer 5’->3’ |
| --- | --- | --- |
| IL-18Rα | CTGGAAGAGCTGTTGTTGAGGAG | GAGAGTGGACTCCATGAAGCA |
| IL-12R | ATACAGTTCCGGACACCTGGAAG | CTATGCCCCCTGGAGATGGA |
| IL-7Rα | ATGGAGACTTTGACGACGCA | GTGGATGGACCTCAGCACTT |
| CCR9 | TTCCTCCCGCCATTGTACTG | TCGTGTTCATCGTGGGTGC |
| CCR5 | TGCCCCCACTCTACTCACT | ATCTACCTGCTCAACCTGGC |
| CXCR6 | CTATGCAGGCATCCACGAGT | TGGGTCTTTGGCAGCATCAT |
| PLZF | CTTCCTGACAACGAGGCAGT | TGGCTGTCTTCTGTCTGCTG |
| T-bet | CCAACCAGTATCCCGTTCCC | GTTTCTACCCCGACCTTCCC |
| RORC | AAGTGGTGCTGGTCAGGATG | CAACGCTGACAACCACACAG |
|  | External reverse primer 5’->3’ | Internal reverse primer 5’->3’ |
| IL-18Rα | CTCCACATGGCTTGACTGTTT | AGCTTCAGTGATTGAGGCAAGA |
| IL-12R | TGCATGGTGGTCCTATTGGC | CCAGAGTTGTTCCCAAGGCA |
| IL-7Rα | CCCTCACGGTAGGTGACTCT | TCTTTCCTCCAAGCTTCACACA |
| CCR9 | GGCTGTACAGGAGTTCTGGG | CCCAGAAAGGAAGGGTGACA |
| CCR5 | GCGTCTGACGATGTGCTTTC | TGGATGGAAAGTGAGGGCTG |
| CXCR6 | GGTCATGCTGTGGTACTCCC | GTGGCAAGGAGACTAGCAGG |
| PLZF | TGCAGATGGTGCACTGGTAG | TTGCACTCGCTGCAGATGTA |
| T-bet | CGCCTTCGCTTAGAGTCTCC | GGGCTGAGGGCAGAAATGTA |
| RORC | GCTTTTCCACGTGCTGACTG | TGATGAAAGGCCAGCTCCAG |
